# Toll-Like Receptor Signaling Pathways—Therapeutic Opportunities

**DOI:** 10.1155/2010/781235

**Published:** 2010-10-17

**Authors:** Jiankun Zhu, Chandra Mohan

**Affiliations:** ^1^The Department of Internal Medicine (Rheumatology), University of Texas Southwestern Medical School, Dallas, TX 75390, USA; ^2^Department of Internal Medicine/Rheumatology, UT Southwestern Medical Center, 5323 Harry Hines Boulevard, Dallas, TX 75390-8884, USA

## Abstract

Toll-like receptors (TLRs) are transmembrane proteins acting mainly as sensors of microbial components. Triggering TLRs results in increased expression of multiple inflammatory genes, which then play a protective role against infection. However, aberrant activation of TLR signaling has a significant impact on the onset of cancer, allergy, sepsis and autoimmunity. Various adaptor proteins, including MyD88, IRAKs, TIRAP, TRIF, and TRAM, are involved in specific TLR signaling pathways. This article reviews the role of these molecules in TLR signaling, and discusses the impact of this pathway on various disease scenarios. Given their important role in infectious and non-infectious disease settings, TLRs and their signaling pathways emerge as attractive targets for therapeutics.

## 1. Introduction—The TLR Pathway

The immune system consists of two closely related systems known as the innate and adaptive immune systems. The adaptive immune system responds to specific “nonself” antigens and generates immunological memory. In contrast, the innate immune system provides an immediate first line of defense against a diverse repertoire of invading microbial pathogens. The key components of innate immunity, cognate pattern recognition receptors (PRRs), are considered to act as sentinels against both invading organisms bearing pathogen-associated molecular patterns (PAMPs) and damage-associated molecular pattern molecules (DAMPs). Toll-like receptors (TLRs) are good examples of these receptors [1]. Because of their wide-ranging impact upon both innate and adaptive immunity in several disease settings, TLRs and their signaling pathways emerge as attractive therapeutic targets. This review summarizes the main players in innate immune signaling and highlights possible drug targets in various disease settings.

Innate immune responses are triggered mainly by a spectrum of “danger” signals referred to as PAMPs and DAMPs. PAMPs are exogenous molecules derived from both pathogenic and nonpathogenic microbes. In contrast, the vast majority of DAMPs are endogenous molecules released from dying host cells molecules upon cellular stress or tissue damage [2–5]. The TLRs are a family of evolutionarily conserved PRRs that play a key role in sensing the microbial world. Different TLR members are reported to recognize and respond to different PAMPs and some endogenous DAMPs [6], thus initiating innate immune responses and priming antigen-specific adaptive immunity, both in infectious and noninfectious disease scenarios. 

**Figure 1:**
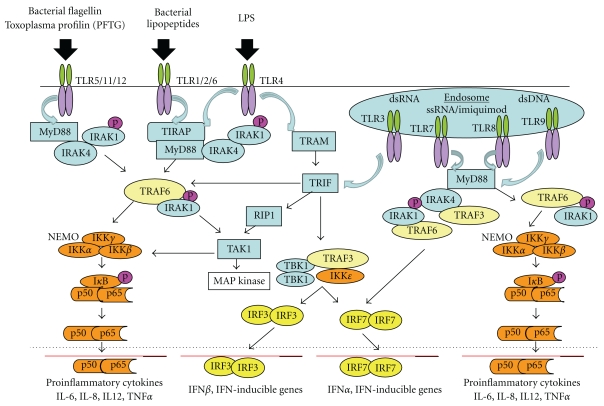
The TLR signaling pathway and downstream effector molecules. Depicted are key TLR molecules, their signaling adaptors and downstream mediators that are essential for TLR signaling and function. The specific molecules in this network that are presently being interrogated as potential therapeutic targets include TLR7/9, MyD88 and IRAK1/4, as discussed in the text.

TLRs are type I transmembrane glycoproteins which are structurally characterized by extracellular leucine-rich repeats (LPRs) and Toll/IL-1 receptor (TIR) signaling domains. The first TLR to be characterized was TLR4 and the family has now been expanded to include 10 members in humans and 12 members in mice [2, 3]. Apart from the highly conserved pathogenic components of bacteria, viruses, fungi, and parasites, TLRs can also be activated by endogenous ligands such as chromatin-IgG complexes [4]. Various TIR domain-containing adaptors such as MyD88, TIRAP, TRIF, and TRAM become engaged or activated upon ligation of TLRs, as diagrammed in Figure 1. TLR1, TLR2, TLR4, and TLR6 recruit TIRAP, which serves as an adaptor between the TIR domain of TLRs and MyD88, while TLR5, TLR7, TLR9, and TLR11 can recruit MyD88 directly. Binding of TLR3 and TLR4 ligands results in the recruitment of TRIF. However, the recruitment of TRIF by TLR4 needs the participation of TRAM (Figure 1). The recruitment of these adaptors triggers a cascade of signaling molecules and ultimately activates the transcription factors, NF-*κ*B and IRFs (Figure 1). These transcription factors induce the expression of various inflammatory cytokines, type I interferons, and chemokines. NF-*κ*B is a central regulator of immune responses involved in cell proliferation and survival and induces the expression of many cytokine and chemokine genes including IL-2, IL-6, IL-12, MCP-1, and TNF-*α* [1]. 

Except for TLR3, all TLR ligation events recruit the adaptor MyD88, followed by the IRAK family of protein kinases, leading to the activation of TRAF6. TRAF6 induces the activation of TAK1 through K63-linked polyubiquitination. NF-*κ*B or AP-1 is then activated by the IKK complex or MAP kinases, respectively (Figure 1). On the other hand, TLR3 initiates a TRIF-dependent pathway that dictates the expression of inflammatory cytokines and type I IFNs via two independent pathways. The N-terminal domain of TRIF interacts with TRAF6 while the C-terminal domain of TRIF interacts with RIP1 and activates TAK1, both of which can activate NF-*κ*B, resulting in the expression of inflammatory cytokines [5]. TLR3 engagement induces the expression of type I IFNs via IRF3 [7]. IRF3 is phosphorylated and activated by the IKK-related kinase, TBK1, facilitated by the recruitment of TRAF3 [8]. TLR7 and TLR9 engagement induces the secretion of inflammatory cytokines through the activation of NF-*κ*B via MyD88. However, TLR7 and TLR9 can also induce the expression of type I IFNs through the activation of IRF7, which is phosphorylated by IRAK1, IRAK4, or IKK*α*, and then translocated to the nucleus where it induces the transcription of IFN-*α* (Figure 1) [9].

As is evident from Figure 1, the IRAK family of molecules plays a pivotal role in mediating almost all TLR-mediated functions. The IRAK family has four members: IRAK1, IRAK2, IRAKM, and IRAK4. All IRAK family members contain an amino-terminal death domain and a serine-threonine kinase domain. IRAK4 is known to be essential for TLR-IL-1R-mediated cellular responses. After TLR ligation, IRAK4 phosphorylates IRAK1 [10]. The IRAK1 gene is composed of 14 exons and is located on the X chromosome [11]. IRAK1 is the first member of this kinase family that was identified as a key component of the IL-1R signaling pathway and plays a specific and essential role in IFN-*α* induction downstream of TLR7 and TLR9 engagement. IRAK1 deficiency abrogates the production of IFN-*α*, IL-6, IL-12, and TNF-*α* when stimulated with TLR7 or TLR9 ligands [12, 13].

## 2. TLR Signaling and Disease

TLRs constitute a primary defense mechanism in both infections and some noninfectious disease settings in mammals. Activation of TLRs and the MyD88 signaling pathway plays a protective role during infection with several pathogens, including protozoan parasites [14] and pyogenic bacteria [15]. Patients with autosomal recessive MyD88 deficiency have been reported to suffer from life-threatening, often recurrent pyogenic bacterial infections. Interestingly, however, their clinical status improved in later life, alluding to the compensatory effect of adaptive immunity [15]. Consistent with their vital role in fighting infections, downregulation of TLR-related molecules or signaling has been associated with sepsis and autoimmune disease, as discussed below. On the other hand, upregulation of these molecules has been linked to cancer, allergy, other autoimmune diseases and immune abnormalities in HIV [16, 17], as detailed below. 

Sepsis is a serious medical condition that is characterized by a systemic state and the presence of a known or suspected, which can lead to rapid tissue damage. The recognition of LPS by TLR4 during an acute Gram-negative bacterial infection plays a key role in the pathogenesis of sepsis. Weighardt and colleagues found that MyD88-deficient mice were protected from developing sepsis in a polymicrobial septic peritonitis disease model [18]. In the IRAK4-deficient model, it was found that mice were severely impaired in their responses to viral and bacterial challenge [19]. Recently, Ferwerda and coworkers reported that polymorphisms in human Mal/TIRAP allele showed association to the pathogenesis of sepsis [20]. On the other hand, others have provided proof that Mkp-1, a key negative regulator of TLR-induced inflammation, plays a critical role in the inhibition of innate immunity during Gram-negative bacteria sepsis [21, 22]. MyD88 also plays a pivotal role in infectious diseases, as demonstrated by Naiki et al. in another model of bacterial pneumonia [23]. Collectively, these studies revealed that the TLR pathway plays a pivotal role in the pathogenesis and control of sepsis.

The importance of TLR expression and function in cancer cells and its association with tumorigenesis and tumor progression have recently been examined. TLR4 expression is linked to several cancers such as gastric cancer and human epithelial ovarian cancer. Huang et al. reported that TLR4 was expressed in murine tumor cells and that the activation of TLR4 in these cells by LPS induced the expression of various soluble factors including interleukin-6, inducible nitric oxide synthase, interleukin-12, B7-H1, and B7-H2 and rendered tumor cells resistant to CTL attack. The factors in LPS-stimulated tumor cell supernatants also had the capacity to inhibit T cell proliferation and natural killer cell activity [24]. Kelly et al., have reported that human epithelial ovarian cancer cells (EOC) ubiquitously express TLR4 and that the proliferation of EOC cells or enhanced cytokine/chemokine production by EOC cells was dependent upon expression of MyD88 [25]. MyD88-dependent signaling controls the expression of several key modifier genes in tumorigenesis and has a critical role in both spontaneous and carcinogen-induced tumor development [26]. Others have reported that gene expression differences in the TLR gene cluster (TLR10-TLR1-TLR6) were associated with a statistically significant reduced risk of prostate cancer [27]. 

Functionally active TLR9 is also known to be expressed on several human tumors, including lung cancer, Burkitt lymphoma, cervical neoplasia, breast cancer, and prostate cancer [28–32]. Although the role of TLR9 in the development of these cancers is not fully understood, TLR9 appears to be playing an important modulatory role in several of these tumors [32–35]. Based on these findings, several TLR9 agonists, including CpG oligonucleotides, are currently in development for the treatment of cancer. These agents have the capacity to upregulate the innate immune response, as well as a humoral immune response against the tumor, as reviewed elsewhere [36].Thus, CpG-ODN can activate plasmacytoid DCs (pDCs) to secrete type I interferon (IFN) and also promote the expression of costimulatory molecules such as CD80 and CD86, on the DCs as well as on TLR9-expressing tumors. This can then lead to the secretion of various cytokines/chemokines, and also activate natural killer (NK) cells, T_H_1 cells, and cytotoxic T lymphocytes (CTLs). Engaging these diverse mechanisms, TLR9 agonists have been found to be therapeutically effective in various cancer trials [36–40]. 

The link between TLR and autoimmune diseases has also become apparent. It was reported that inhibition of TLR4 suppressed the severity of experimental arthritis and resulted in lower IL-1 expression in arthritic joints [41]. Roelofs found that a TLR4 variant (Asp299Gly) could reduce its potency to mediate signaling in rheumatoid arthritis patients [42]. A TLR2 R753Q polymorphism has been reported to be more prevalent among reactive arthritis patients [43]. Likewise, polymorphisms in TLR2 and TLR4 may also be important in the pathogenesis of SLE in both patients [44] and mouse models [45]. Moreover, TLR7 has been suggested to be functionally involved in autoantibody production, and hence closely linked to the pathogenesis of SLE [46]. Indeed, the translocation of the X-linked TLR7 gene to the Y-chromosome has been documented to facilitate the development of fatal lupus in mice with numerous immunological aberrations, hence constituting disease genes for murine lupus [47, 48].

The impact of MyD88 and IRAK1 deficiency on autoimmunity has been quite well documented. Harada and coworkers reported that MyD88-deficiency protected MRL/lpr mice from the development of autoimmune nephritis, marked by lower levels of serum anti-double-stranded DNA (anti-dsDNA) antibodies and reduced cytokines, including interferon-*α*, interleukin-12, IL-6, and IFN-*γ* [49]. Similarly it was reported that MyD88-deficient mice were completely resistant to experimental autoimmune uveitis (EAU) and had reduced Th1, but not Th2, responses upon immunization with retinal Ag [50]. In all of the above studies of sepsis, cancer, and autoimmunity, a caveat should be kept in mind when interpreting findings derived using MyD88-deficient mice. It has been documented that MyD88-deficient mice show a loss of IL-1- and IL-18-mediated functions indicating that the phenotypes observed in Myd88-deficient mice might arise in part because of defects in signaling downstream of the IL-1 and Il-18 receptors [51]. 

Autoimmunity also appears to be contingent upon the expression of another TLR signaling molecule, IRAK1. It has been reported that IRAK1-deficient mice are resistant to experimental autoimmune encephalomyelitis (EAE), exhibiting little or no inflammation in the central nervous system [52]. IRAK1-deficient mice exhibited impaired Th1 cell development with minimal IFN-*γ* secretion during disease induction following immunization with myelin antigen [52]. The authors suggested that the mechanism underlying this observation might relate to an impaired adjuvant effect on antigen presenting cells as a result of suboptimal TLR activation. Our recent study has revealed that the IRAK1 gene was highly associated with both adult- and childhood-onset SLE [53]. Using the IRAK1-deficient mice, we found that in mice bearing the lupus susceptibility loci, *Sle1* or *Sle3*, IRAK1 deficiency abrogated all lupus-associated phenotypes, including IgM and IgG autoantibodies, lymphocytic activation, and renal disease. In addition, the absence of IRAK1 reversed the dendritic cell “hyperactivity” associated with lupus [53]. 

Heme oxygenase-1 (HO-1), a key cytoprotective, antioxidant, and anti-inflammatory molecule, was reported to be involved in the activation of IRF3 after TLR3 or TLR4 stimulation, or viral infection. HO-1 is necessary for the expression of primary IRF3 target genes encoding RANTES, IP-10, and MCP-1. In the experimental autoimmune encephalomyelitis model, mice with myeloid-specific HO-1 deficiency developed severe disease correlating with hyperactive antigen-presenting cells, enhanced infiltration of Th17 cells, and nonregressing myelin-specific T cell reactivity [54]. Interestingly, IFN-*β* could alleviate these defects. The overproduction of IFN-*α* and IFN-*α*-dependent genes, such as IRF5, are additional factors that play a key role in the pathogenesis systemic lupus erythematosus [42].

## 3. Potential Therapeutic Targets in the TLR Signaling Pathway

Since the bulk of the data suggests that the TLR pathway plays a key role in multiple pathogenic processes, the targeting of either the TLRs themselves or the signals they generate is of great interest, as reviewed by O'Neill [55]. As MyD88 is clearly involved in infectious disease, cancer, and autoimmune diseases, it is obviously an attractive target for intervention in these diseases. Loiarro et al. have reported a MyD88 inhibitor ST2825, a heptapeptide analog specifically designed to inhibit MyD88 dimerization, which could control TLR mediated inflammatory responses [56]. ST2825 is reported to inhibit MyD88 dimerization in co-immunoprecipitation experiments, and is specific for homodimerization of the TIR domains but does not affect homodimerization of the death domains. They tested ST2825 in experimental animal models of autoimmune, and inflammatory diseases, such as lupus, inflammatory bowel disease, and multiple sclerosis, and found that ST2825 could interfere with the recruitment of IRAK1 and IRAK4 by MyD88, resulting in the inhibition of IL-1*β*-mediated activation of NF-*κ*B and IL-6. They also observed that ST2825 suppressed B cell proliferation and differentiation into plasma cells in response to CpG-induced activation of TLR9. Bartfai et al. have also designed a low molecular weight MyD88 mimic, “Compound 4a”, which could interfere with the interaction between MyD88 and IL-1R at the TIR domains [57]. Compound 4a inhibited IL-1*β*-induced phosphorylation of the mitogen-activated protein kinase p38 in EL4 thymoma cells and in freshly isolated murine lymphocytes, and significantly attenuated IL-1*β*-induced fever response in vivo. These data suggest that MyD88 might be a potential target for pharmaceutical usage in several disease settings where increased TLR signaling is driving disease pathogenesis.

Modulation of another signaling molecule in the TLR pathway, IRAK4, has also been suggested to be an attractive therapeutic approach for the treatment of cancer, autoimmune and inflammatory diseases. A series of small-molecule compounds that inhibit IRAK4 has been developed by Wang et al. [58]. However, the effectiveness of these IRAK4 inhibitors in vitro and in vivo remains to be documented. Another group has reported that a dual inhibitor of IRAK1 and IRAK4 (RO0884) could efficiently inhibit both IL-1*β* and TNF-*α* induced p38 MAP kinase, c-Jun N-terminal kinase activation, and IL-6 production in human cells [59]. Through silencing IRAK1 or IRAK4 gene by siRNA, they also found that IRAK4 was essential for response to IL-1*β* but not to TNF-*α*, whereas IRAK1 was essential for cellular responses to TNF-*α* but not IL-1*β*. These data suggested that inhibition of both IRAK1 and IRAK4 kinases would be necessary to block proinflammatory cytokine production [59]. Collectively, these findings raise hope that IRAK1/IRAK4 inhibitors may be of therapeutic benefit in autoimmune and inflammatory diseases. 

Administration of synthetic oligodeoxynucleotides with immunoregulatory sequences (IRS) that specifically inhibit both TLR7 and TLR9 signaling (IRS 954) has been shown to decrease the production of IFN-*α* by human PDCs after stimulation with DNA or RNA viruses [60]. It has been reported that (NZB×NZW) F1 mice showed decreased serum levels of antinuclear autoantibodies, proteinuria, and glomerulonephritis, with increased survival upon IRS 954 treatment [61]. Another oligodeoxynucleotides inhibitor IRS 661, which is specific to TLR7, had the capacity to significantly reduce disease in MRL/lpr mice, particularly the weights of spleen and lymph nodes and serum levels of TNF-*α* as compared with saline-treated control [62]. Injection of IRS 661 but not IRS 954 can significantly decreased anti-dsDNA and anti-Sm antibodies in lupus-prone mice. Most interestingly, both IRS 661 and IRS 954 could be detected in the kidneys and were found to be taken up by glomerular cells, tubular epithelial cells, and intrarenal macrophages. The mRNA levels of inflammatory chemokines CCL2 and CCL5 and their respective chemokine receptors CCR2 and CCR5 were also decreased in the kidneys. Consistent with these findings, the activity and chronicity scores for antibody-mediated nephritis in IRS 661- or IRS 954-treated mice were also reduced significantly [62]. Besides lupus, these inhibitors have also shown efficacy in other autoimmune diseases including arthritis and multiple sclerosis [63–65]. Based on a recent report suggesting that SLE in patients is also contingent upon TLR signaling [66], antagonists of this pathway are expected to confer therapeutic benefit in patients as well.

## 4. Conclusion

TLR molecules and their downstream signaling pathways play a critical role in activating innate immune cells and selected cell types in the adaptive arm of the immune system. Given that this pathway is aberrantly expressed or activated in several diseases, this axis constitutes an attractive target for therapeutic intervention. There is mounting evidence documenting that the crippling of this pathway at the level of TLR, MyD88, or IRAK1/4 may confer therapeutic efficacy in autoimmunity and auto-inflammatory diseases. On the other hand, the total crippling of these pathways may compromise immune defense against invading infections and immune surveillance against cancers. In fact, agonists of these pathways appear to be useful in some of these disease settings. Hence there is a need to carefully select the therapeutic target in the TLR signaling cascade, and closely regulate the degree of pathway activity so as to attain the desired therapeutic end-point. Like many other molecules in biology, the TLR signaling axis emerges as yet another “double-edged sword”.
